# Detection of Bone Metastases in Initial Staging of Orbital Embryonal Rhabdomyosarcoma by Fluorodeoxyglucose Positron Emission Tomography/Computed Tomography

**DOI:** 10.4274/MIRT.20.07

**Published:** 2011-04-01

**Authors:** Pelin Ozcan Kara, Gonca Kara Gedik, Oktay Sari

**Affiliations:** 1 Selcuk University Selcuklu Medical Faculty, Department of Nuclear Medicine, Konya, Turkey; 2 Selcuk University Meram Medical Faculty, Department of Nuclear Medicine, Konya, Turkey

**Keywords:** Rhabdomyosarcoma; FDG-PET/CT; initial staging; bone metastases

## Abstract

Rhabdomyosarcoma is the most common form of soft tissue sarcoma in young children. In soft tissue sarcomas, isolated metastases are seen in the lung, soft tissue, and bone. The optimal management of these tumors depends on the site, size, and grade of the local growth, and accurate staging of the disease when first seen. Although detection of the primary site of disease is usually accomplished well with conventional techniques, the performance of fluorodexyglucose (FDG) positron emission tomography/computed tomography (PET/CT) may be useful to determine metastases that are not clinically evident. We describe a case of early detection of distant metastases by FDG PET/CT in a young patient diagnosed with orbital embryonal rhabdomyosarcoma.

**Conflict of interest:**None declared.

## CASE REPORTS

**Figure 1:**
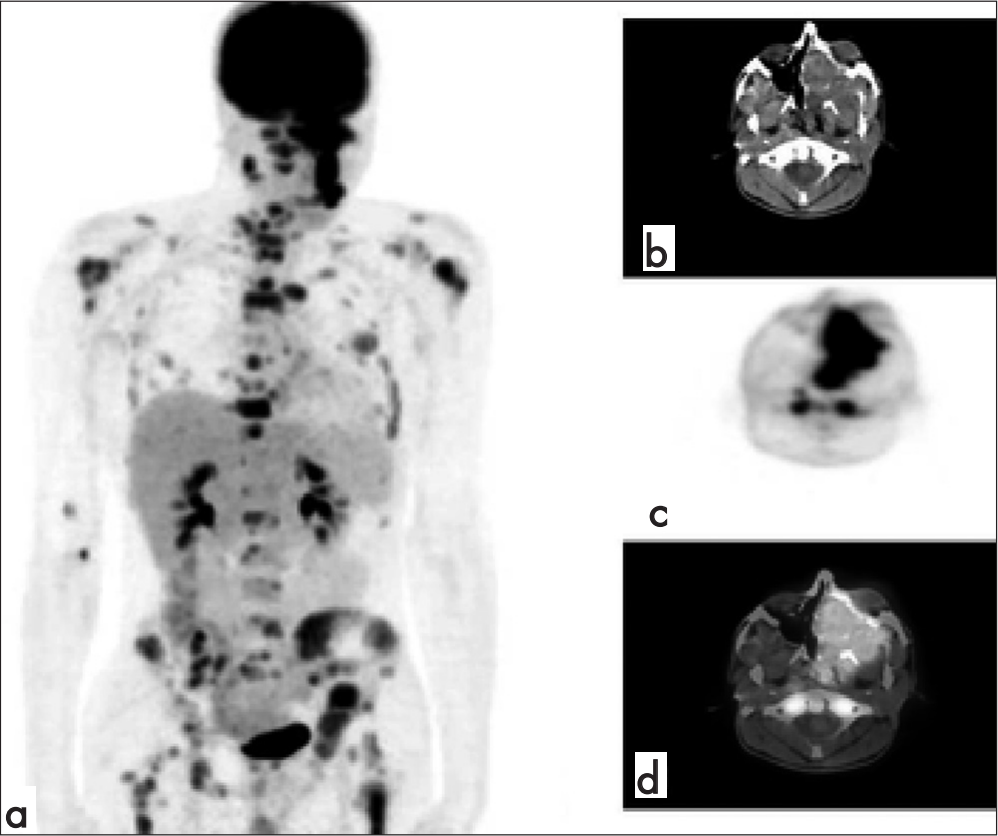
MIP image (a) and transaxial images (b,c,d) of PET/CT revealed the primary lesion (SUVmax: 9.82) and bilateral cervical lymph nodes (SUVmax 9.44), pelvic bones, bilateral proximal femora, bilateral humeri, scapula, sternum, clavicles, ribs, and vertebrae

A 17-year-old boy presented to our department with dyspnea and diplopia lasted for approximately one month. MRI demonstrated 6 x 4 x 4 cm mass with irregular contour in the orbital region. On MRI, one left cervical and one submandibular lymph node were also detected. Embryonal rhabdomyosarcoma (RMS) was diagnosed by biopsy. To better define the lesion prior to surgery and in a search for distant metastases, we performed a combined FDG PET/CT whole-body scan. PET/CT revealed the primary lesion with irregular contours. Maximum calculated standardized uptake value (SUVmax) of the lesion was 9.82 PET/CT also demonstrated bilateral cervical and submandibular lymph nodes showing intense FDG accumulation (SUVmax 9.44). Additionally, there were pathological accumulations in pelvic bones, bilateral proximal femora, bilateral humeri, scapula, sternum, clavicles, ribs, and vertebrae (Figure 1). The patient could not undergo complete resection because of the local invasion on computed tomography and magnetic resonance imaging and distant metastases. Chemoradiotherapy was initiated. In our patient, FDG-PET combined with simultaneous low-dose CT transmission tomography, permitted the detection of distant metastases that was not clinically evident. PET/CT also showed pathological contralateral cervical lymph nodes that were not seen on MRI. The patient was upstaged on FDG PET/CT. The orbit is one of the most common sites for RMS. Most of the patients with orbital sarcoma present with localized disease. Patients presenting metastatic disease at initial diagnosis are uncommon. However, bone marrow involvement counts among the most common metastases, especially in the presence of extensive local invasion (1). Embryonal RMS is the most common histology in patients with non-metastatic orbital sarcoma. In Stage 4 cases, embryonal RMS was found in about half of the cases (2). There are few reports about the efficacy of FDG PET/CT in the localization and detection of soft tissue sarcomas (3,4). In a study by Tateishi et al (5), 11 of the 35 patients had distant metastases detected by FDG PET/CT, which were not identified by conventional radiologic evaluation. FDG PET/CT was found more accurate than conventional imaging regarding clinical staging and re-staging of patients with rhabdomyosarcomas. Although, overall likelihood of bone involvement is low in orbital embryonal RMS at initial diagnosis, FDG PET/CT may be useful in patients with a higher likelihood of distant metastases.
